# Molecular Characterization of a Prevalent Ribocluster of Methicillin-Sensitive *Staphylococcus aureus* from Orthopedic Implant Infections. Correspondence with MLST CC30

**DOI:** 10.3389/fcimb.2016.00008

**Published:** 2016-02-16

**Authors:** Lucio Montanaro, Stefano Ravaioli, Werner Ruppitsch, Davide Campoccia, Giampiero Pietrocola, Livia Visai, Pietro Speziale, Franz Allerberger, Carla Renata Arciola

**Affiliations:** ^1^Research Unit on Implant Infections, Rizzoli Orthopaedic InstituteBologna, Italy; ^2^Department of Experimental, Diagnostic, and Specialty Medicine, University of BolognaBologna, Italy; ^3^Division of Human Medicine, Austrian Agency for Health and Food SafetyVienna, Austria; ^4^Unit of Biochemistry, Department of Molecular Medicine, University of PaviaPavia, Italy; ^5^Molecular Medicine Department, Center for Health Technologies (CHT), UdR INSTM, University of PaviaPavia, Italy; ^6^Department of Occupational Medicine, Toxicology and Environmental Risks, S. Maugeri FoundationIRCCS, Pavia, Italy

**Keywords:** methicillin-sensitive *Staphylococcus aureus*, orthopedic implant infections, multilocus sequence typing, spa-typing, virulence factors

## Abstract

*Staphylococcus aureus* is the leading etiologic agent of orthopedic implant infections. Here a ribocluster of 27 *S. aureus* strains underwent further molecular characterization and subtyping by multilocus sequence typing (MLST) and *spa*-typing. This cluster had been detected by automated ribotyping (with the EcoRI restriction enzyme) of 200 *S. aureus* isolates from periprosthetic infections of patients who underwent revision at the Rizzoli Orthopaedic Institute. The ribocluster, consisting of *agr* type III strains, with a 74% co-occurrence of bone sialoprotein-binding (*bbp*) and collagen-binding (*cna*) genes, lacked *mecA* and IS*256*, and exhibited a high prevalence of the toxic shock syndrome toxin gene (*tst*, 85%). Strains' relatedness was analyzed by BURP and eBURST. Two predominant *spa* types, t012 (32%) and t021 (36%), and one predominant sequence type, ST30 (18/27, 67%) were identified: a *S. aureus* lineage spread worldwide belonging to MLST CC30. Two new sequence types (ST2954, ST2960) and one new spa type (t13129) were detected for the first time. Interestingly, the 27-strain cluster detected by ribotyping corresponded exactly to MLST CC30, the sole CC identified by eBURST.

## Introduction

*Staphylococcus aureus* is the leading etiologic agent of orthopedic implant-associated infections (Arciola et al., 2005a; Montanaro et al., 2011; Rao et al., 2011; Tande and Patel, 2014). At present, the properties that allow particular *S. aureus* clones to prevail and become epidemic are not known. Infections related to orthopedic implants occur in ~1.5% of cases (Montanaro et al., 2011; Tsaras et al., 2012; Tande et al., 2014). The high number of primary and revision arthroplasties renders these infections significant in terms of morbidity, mortality, and economic consequences (Montanaro et al., 2011). The potential to colonize host periprosthetic tissues and to cause severe disease differs among clonal lineages, a feature that is attributed to the absence or presence of different virulence factors and to the levels at which they are produced (Li et al., 2009). In orthopedic implant infections, the first microbial adhesion to a biomaterial coated by host extracellular proteins is mediated by *Microbial Surface Components Recognizing Adhesive Matrix Molecules* (MSCRAMMs; Speziale et al., 2009; Foster et al., 2014). Among these, bone sialoprotein-binding protein (Bbp) and collagen adhesin (Cna) play a crucial role in the onset of device-related infections (Arciola et al., 2005b; Xu et al., 2005; Campoccia et al., 2009; Vazquez et al., 2011; Post et al., 2014). *S. aureus* can also yield a variety of other virulence factors, such as Panton-Valentine leukocidin (PVL), often associated with necrotizing pneumonia and skin infections (Rasigade et al., 2011), toxic shock syndrome toxin (TSST), a super-antigenic toxin more common in MSSA lineages (He et al., 2013), and the insertion sequence IS*256*, widespread in genomes of multi-resistant staphylococci (Byrne et al., 1989; Depardieu et al., 2007; Schreiber et al., 2013).

*S. aureus* has evolved into many clones, some of which are rare and referred to as “sporadic,” and others, with a worldwide prevalence, can be defined “epidemic.” The evolutionary success of different clones of the same microorganism indicates the acquisition of new traits that either boost their virulence or favors their adaptability in the “race for the surface” between bacteria and eukaryotic cells in particular niches of infections, such as the biomaterial/tissue interface (Gristina et al., 1988-1989; Feng et al., 2008; Montanaro et al., 2011).

The clonality of *S. aureus* was initially revealed by multilocus enzyme electrophoresis (MLEE)-typing and pulsed field gel electrophoresis (PFGE)-typing. Multilocus sequence typing (MLST), based on the profile of alleles at seven loci of housekeeping genes, and *spa*-typing, based on the variable X-region of the staphylococcal protein A gene, have confirmed the highly clonal structure of *S. aureus* (Shopsin et al., 1999).

This study was aimed at thoroughly investigating the genetic background of a cluster of 27 *S. aureus* strains (Campoccia et al., 2009). This cluster was identified when ribotyping 200 *S. aureus* isolates obtained from patients undergoing revision at the Rizzoli Orthopaedic Institute (IOR) for periprosthetic infections (Campoccia et al., 2009). The ribocluster strains were analyzed by MLST and by *spa* typing in order to establish whether they belonged to a single clonal complex, and ultimately to ascertain if the most prevalent cluster identified by riboprinting from a collection of orthopedic implant infections corresponded to some known epidemic clonal complex, as the historical CC30 and its lineages (see Table 1S for a biographical sketch of CC30). To this end, the resulting sequences were analyzed by BURP and eBURST algorithms. In the present study, isolates were further characterized by assaying their antibiotic-resistances and checking for the presence of *mecA* for methicillin-resistance. The search for IS*256*, Panton-Valentine *pvl* gene, and TSST gene *tst* provided additional information for the epidemiological and pathogenetic profiles.

## Materials and methods

### *S. aureus* ribocluster

A ribocluster of 27 *S. aureus* strains was utilized in this study. Three ribogroups form this ribocluster: *cra*-119-S-8, *cra*-138-S-2, and *cra*-53-S-7. These ribogroups had been identified by an automated RiboPrinter® and then recognized as a unique ribocluster when ribotyped among 200 *S. aureus* isolates from infected prostheses observed at the IOR of Bologna (Campoccia et al., 2009). The automated RiboPrinter® is prone to categorizing the strains that diverge only at the level of bands with molecular weight greater than 50 Kbp as belonging to different ribogroups, and expert supervision is necessary (Brisse et al., 2002). We designated all 27 strains as a single large cluster (Campoccia et al., 2009).

Clinical isolates came from revision of surgical wounds and treatment of infected prostheses of the following categories: external fixation devices (EF), internal fixation devices (IF), knee arthroprostheses (K), and hip arthroprostheses (H).

Staphylococcal species identification was previously performed Api-Staph and/or ID 32 Staph test (BioMérieux, Marcy l'Etoile, France). Following criteria of the Centers for Disease Control and Prevention (CDC) to distinguish community-acquired (CA) and hospital-acquired (HA) infections^1^, the *S. aureus* isolates of this study were categorized as hospital acquired (HA). The strains were stored at −80°C. The study was approved and funded by the Scientific Director of the IOR. All microbiological samples were completely de-identified and stripped of all patient identifying information.

### Bacterial DNA isolation

The chromosomal DNA used as an amplification template was extracted from the bacterial cultures using QIAmp DNA mini kit (Qiagen, GmbH, Hilden, Germany), according to the manufacturer's instruction.

### Detection of *mecA, femA, pvl*, IS*256*, and *tst* genes

PCR conditions and primers used in this study are reported in Table 1.

**Table 1:** **PCR conditions and primers used in this study**.

**Target gene**	**Primer sequences**	**Amplicon size (bp)**	**References**
*mecA*	5′-TGGCTATCGTGTCACAATCG-3′ 5′-CTGGAACTTGTTGAGCAGAG-3′	310	Vannuffel et al., 1995
*femA*	5′-CTTACTTACTGGCTGTACCTG-3′ 5′-ATGTCGCTTGTTATGTGC-3′	686	Vannuffel et al., 1995
*Pvl* (*lukS*-PV/*lukF*-PV)	5′-ATCATTAGGTAAAATGTCTGGACATGATCCA-3′ 5′-GCATCAASTGTATTGGATAGCAAAAGC-3′	433	Lina et al., 1999
IS*256*	5′-AGTCCTTTTACGGTACAATG-3′ 5′-TGTGCGCATCAGAAATAACG-3′	762	Gu et al., 2005
*Tst*	5′-ATGGCAGCATCAGCTTGATA-3′ 5′-TTTCCAATAACCACCCGTTT-3′	349	Jarraud et al., 1999

*The presence of genes was tested by amplification of the respective gene-fragments using 10 μl RedTaq® ReadyMix™ PCR Reaction Mix (Sigma, St Louis, MO), 1 μl gDNA, and 10 pmol/μl primer. Amplification conditions were as follows: an initial step of 5 min at 95°C, 40 cycles each of 30 s at 95°C, 45 s at 55°C, and 45 s at 72°C, and a final step of 45 s at 72°C*.

### Antibiotic susceptibility

The agar diffusion (Kirby-Bauer) method was utilized to perform the antibiotic susceptibility tests according to Clinical and Laboratory Standards Institute (CLSI) guidelines (NCCLS, 2002). Antimicrobial susceptibility was tested for a panel of 16 antibiotics: oxacillin (OXA), imipenem (IMP), penicillin (PEN), ampicillin (AMP), cefazolin (CFZ), cefamandole (FAM), gentamicin (GEN), amikacin (AMK), netilmicin (NET), tobramycin (TOB), erythromycin (ERY), clindamycin (CLI), chloramphenicol (CHL), trimethoprim–sulfamethoxazole (SXT), ciprofloxacin (CIP), and vancomycin (VAN).

### *spa* sequencing

The polymorphic X, or short sequence repeat (SSR), region of the *S. aureus* protein A gene (*spa*) was amplified by PCR with primers 1113F (5′-TGTAAAACGACGGCCAGT-3′) and 1514R (5′-CAGGAAACAGCTATGACC-3′) according to protocols previously described (Schmid et al., 2013).

Ten microliters of the amplified products were analyzed on 1.5% agarose gels and 5 μl were purified with EXO SAP-IT (GE Health care, Buckinghamshire, GB). Two microliters of the purified amplification products were used for subsequent sequencing using the Big Dye Terminator v3.1 sequencing kit (Applied Biosystems, Carlsbad, CA) and were finally analyzed on ABI Genetic Analyzer 3500Dx (Applied Biosystems).

The chromatograms obtained were analyzed with the Ridom StaphType software (version 1.4; Ridom GmbH, Würzburg, Germany; http://spa.ridom.de/index.shtml) to determine the *spa* type of each isolate^2^. The *spa* types were deduced by the differences in number and sequence of *spa* repeats. Using the BURP algorithm (Ridom GmbH) and the Ridom SpaServer database (Enright et al., 2000), *spa* types were clustered into different clonal complexes (*spa*-CCs) and MLST clonal complexes (CCs) were inferred.

### Multilocus sequence typing

MLST genotyping was performed on all 27 *S. aureus* isolates as described previously by Larsen et al. (2012). The amplification of a portion of seven housekeeping genes (*arc, aroE, glp, gmk, pta, tpi, yqiL*) was performed and then sequenced. The free cross-platform bioinformatics software package Unipro UGENE 1.13^3^ was used to analyze the sequences. Sequence types (STs) were obtained using the MLST database^4^. Using the eBURST v3 algorithm^5^, sequence types (STs) were clustered to assign the clonal complexes (CCs), and assess the population organization and patterns of evolution. *S. aureus* strains with STs differing by one or two housekeeping genes/loci were considered part of a unique clonal complex.

## Results

### MLST analysis

The MLST analysis of the 27 strains of the ribocluster identified 5 distinct STs. ST30, the most prevalent, included 18 strains (67%), ST34 consisted of 5 strains (19%), ST2954 of 2 strains (7%), and ST2960 and ST243 were both represented by a single strain. The allelic profile of each identified ST is reported in Table 2. Sequence type attribution, riboprofiles, genotypic characteristics, and clinical origin of each of the 27 strains investigated are summarized in Table 3.

**Table 2:** **Description of all the STs found in the present study**.

**ST**	MLST allelic profile^*^	***spa* types**	***spa* repeat succession**	***spa* CC**
**ST30 (18)**	2-2-2-2-6-3-2	t018 (2)	**15-12-----16-02-16-02-25-17-24-24-24**	CC021/012
		t093 (1)	**15-12-12-16-02-16-02-25-17-24-24----**	
		t012 (7)	**15-12-----16-02-16-02-25-17-24-24----**	
		t021 (6)	**15-12-----16-02-16-02-25-17-24--------**	
		t1382 (1)	**01**-12-----16-02-16-02-25-----24--------	
		t11956 (1)	15-12-----**23**-----16-02-25-17-24--------	
**ST243 (1)**	2-2**-5-**2-6-3-2	t021 (1)	**15-12-----16-02-16-02-25-17-24--------**	
**ST2960**^a^ **(1)**	2-2-2-2-6**-268-**2	t021 (1)	**15-12-----16-02-16-02-25-17-24--------**	
**ST2954**^a^ **(2)**	2-2-2-2-6-3**-330**	t298 (2)	**15-12-----16-02-------------17-24--------**	
**ST34 (5)**	**8-**2-2-2-6-3-2	t166 (2)	**04-44-33-31-12-16-34-16-12-25-22-34**	CC166
		t369 (1)	**04-44-----31-12-16-34-16-12-25-22-34**	
		t4437 (1)	**04-44-33-31-12-16---------12-25-22-34**	
		t13129^b^ (1)	**04-51----------------34-16 -12-25-22-34**	Singleton

* MLST allelic profile (arc-aroe-glpf-gmk-pta-tpi-yiql); numbers between brackets represent the number of strains;

a newly described ST;

b *newly described spa type; MLST alleles shared with the probable founder of MLST CC30, ST30, are colored in sky blue; the spa sequence repetitions shared with the probable founder of spa CC021/012, t021, appear in blue; the spa sequence repetitions shared with the probable founder of spa CC166, t166, are in red; the spa sequence repetitions of the new spa type t13129 shared with the probable founder of spa CC166 are in green. repeat 23 differs in one base from repeat 16; repeat 01 differs in three bases from repeat 15; repeat 51 differs in three bases from repeat 44*.

**Table 3:**
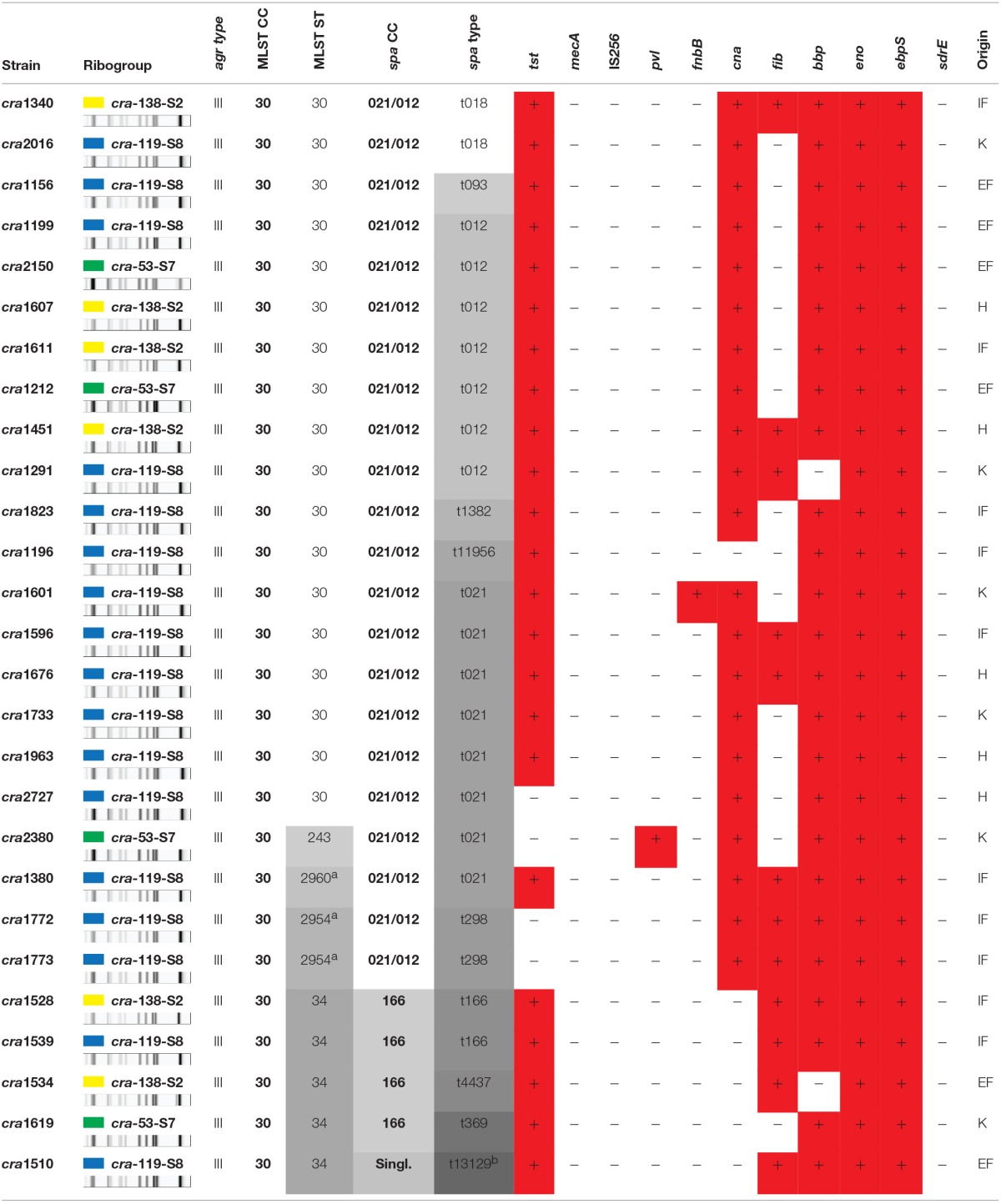
**Detailed genotyping characterization data of the 27 strains**.

*Genes codifying for: eno, laminin-binding adhesin; ebpS, elastin-binding protein; fib, fibrinogen-binding adhesin; can, collagen-adhesin; fnbB, fibronectin-binding protein B; bbp, bone sialoprotein-binding protein; sdrE, serine-aspartate repeat proteins E. K, H = knee, hip arthroprostheses; EF, IF = external, internal fixation systems. ^a^New sequence type (ST), ^b^new spa type found in this study; Singl. = singleton*.

The strains of the studied ribocluster had been previously characterized for their *agr* type (Montanaro et al., 2010) and for the following panel of MSCRAMM genes (Campoccia et al., 2009): *eno, fib, cna, ebpS, fnbB, bbp*, and *sdrE*. Strains were all of the *agr* type III. All 27 strains turned out negative for *mecA*, IS*256*, and *sdrE*, and positive for *eno* and *ebpS* genes.

As shown in Table 4, ST30 was found to consist mainly (86%) of *cna*- and *bbp*-positive strains: only one strain out of 18 (6%) was found to be either *cna*- or *bbp*-negative. Thus, a remarkably high proportion of the ST30 strains exhibited a typical *bbp*-*cna* adhesin co-occurence. The *bbp* gene encoding the Bbp was observed in 93% of the 27 strains (Campoccia et al., 2009), often in association with the *cna* gene detected in 78% of the strains.

**Table 4:** **Genotypic characteristics of the ST, *spa*-CC, and *spa*-types**.

**ST**	***spa* CC**	***spa* type**	***bbp-cna* tandem**	***fib***	***tst***	***agr***	**IS*256***	***mecA***
ST2960 (1)	CC021/012	t021 (8; 36%)	100%	100%	100%	III	neg	neg
ST243 (1)				–	–			
ST30 (18)				33%	83%			
		t012 (7; 32%)	86%	29%	100%			
		Other *spa* types (5; 23%)^a^	80%	20%	100%			
ST2954 (2)		t298 (2; 9%)	100%	100%	–			
ST34 (5)	CC166	t166 (2; 50%)	–	100%	100%			
		Other *spa* types (2; 50%)^b^	–	50%	100%			
	singleton	t13129 (1; 100%)	–	100%	100%			

*Numbers between brackets represent the number of strains and percent frequency*.

a *The remaining spa types are t018 (2), t093, t1382, t11956*.

b *The remaining spa types are t369, t4437*.

All ST30 strains except for *cra*2727 were characterized by the presence of *tst* gene, which was unusually found to be extremely common in this collection of strains. The ST30 *fib*-positive strains were 5 (29%) and *cra*1601 was the only strain carrying the *fnbB* gene (see Table 4). All ST30 strains were found to be *pvl*-negative.

All five ST34 strains were *cna*-negative (100%) in contrast to the remaining strains of the ribocluster, 95% of which were *cna*-positive.

As far as the other minor STs are concerned, the only ST243 strain, *cra*2380, and the two ST2954 strains were found to be *tst*-negative. Apart from these strains belonging to ST243 and ST295, the *tst* gene was generally present among the other strains of the ribocluster and only one strain (cra2727) out of 24 was found *tst*-negative. Further, *cra*2380 was also the only strain of the collection found positive for *pvl*. All three ST2954 and ST243 strains however exhibited the *bbp*-*cna* combination of genes typical of ST30. The single strain of the newly identified ST2960 matched the characteristics of ST30, testing positive to both *bbp* and *cna* and presenting the *tst* gene.

**Figure 1:**
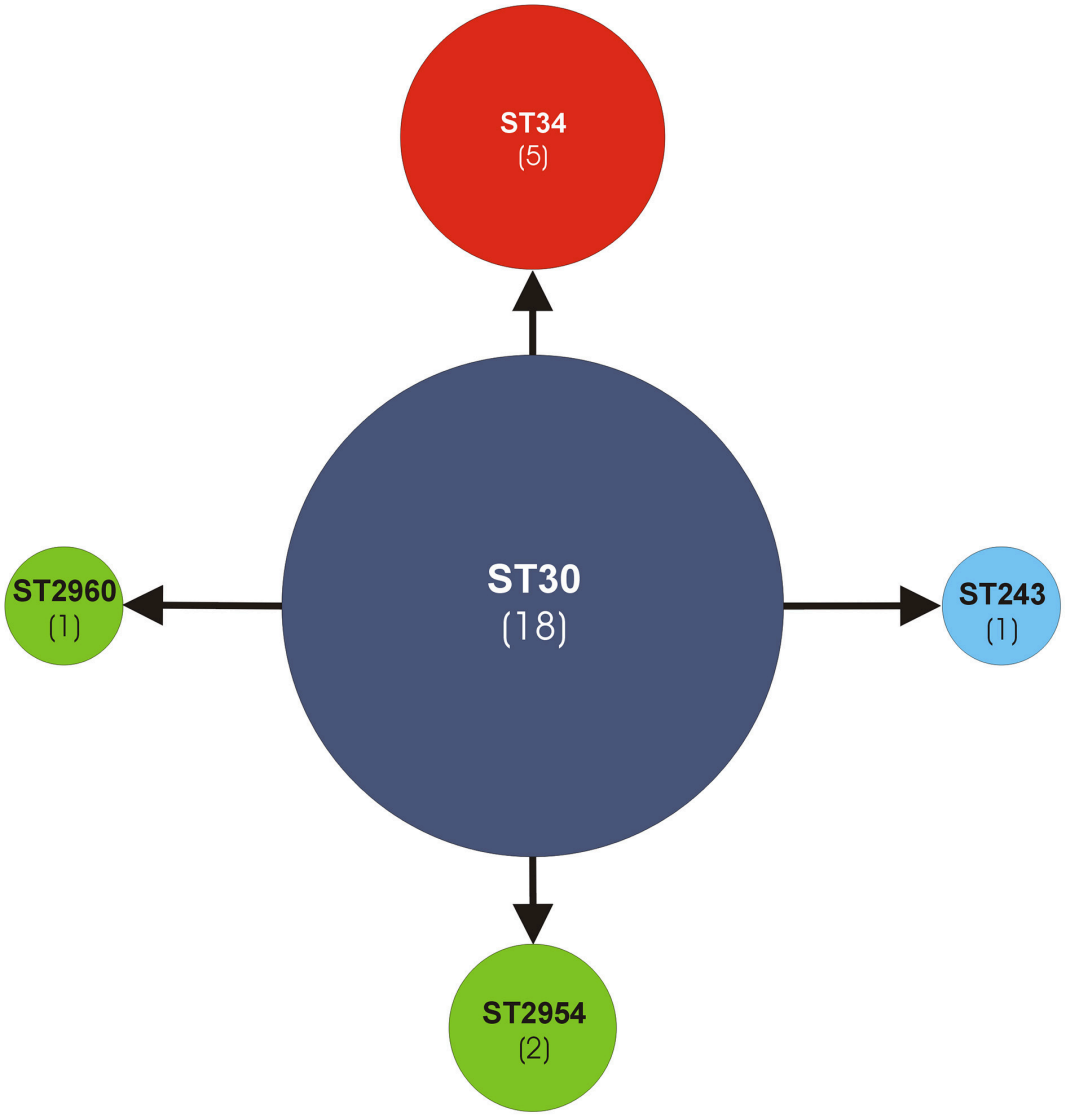
**Analysis of ST allelic profiles of the MLST CC30 by eBURST v3 software**. The sequence type ST30, genotypic founder of the MLST CC30, is represented by a blue circle. All other STs are SLV (single locus variant). The new STs (ST2960, ST2954) are represented by green circles. ST34, a sub-group founder, is represented by a red circle. The other ST243 is represented by a sky-blue circle. In parentheses the number of strains.

Based on the analyses by the eBURST algorithm, the five STs were clustered within the same MLST clonal complex CC30 (Figure 1). ST30 (18/22, 82%) was recognized as the genotypic founder and ST34 (5/22, 23%) as a subgroup founder. The remaining single locus variant (SLV) STs were ST243 (1/22) and the new alleles first discovered in this work, namely ST2954 (2/22) (strain *cra*1772 and strain *cra*1773) and ST2960 (1/22) (strain *cra*1380). Thus, the 27-strain ribocluster was entirely associated with the CC30, confirming the strict kinship of the 27 strains.

### *spa* typing

The *spa* typing analysis revealed 11 distinct *spa* types within the group of 27 strains. The different *spa* repeat sequences specific for each identified *spa* type is reported in Table 2. The *spa* type including the largest number of strains was t021, enlisting 8 out of 27 strains (30%). It was immediately followed by t012, consisting of 7 strains (26%). All the other 9 *spa* types included just 1 or at most 2 strains (representing a frequency of 4–7%) as reported in Table 2. Among these less frequent *spa* types, there was a newly identified *spa* type, t13129 (strain *cra*1510), never described before. The *spa* type attribution of each single clinical strain is reported in Table 3, while the genotypic traits characteristic of the *spa* types are described in Table 4.

BURP analysis of the *spa* types yielded two main clonal complexes, *spa*-CC021/012 (22 strains) and *spa*-CC166 (4 strains), and a singleton (Table 4). The *spa* types in the large *spa*-CC021/012 were: t021 (8 strains out of 22, 36%), t012 (7, 32%), t018 (2, 9%), t298 (2, 9%), t093 (1, 5%), t1382 (1, 5%), and t11956 (1, 5%). Thus, two *spa* types, t021 and t012 together represented up to 68% of the *spa*-CC and 59% of the entire collection. These two *spa* types differed in just one sequence repeat at the end of the repeat sequence succession. The repeat sequence 24 was just in one copy in t021 and double in t012. The two strains of *spa*-CC021/012 with *spa* type t298 were belonging to the ST2954.

Apart from the founder t166, *spa*-CC166 had 2 further *spa* types: t369 and t4437. Discovered in the present study and identified by BURP analysis as a singleton, *spa* type t13129 (strain *cra*1510) exhibited a repeat succession just partially different from that of t166, lacking four repeat sequences (33-31-12-16) and carrying the 51 repeat sequence instead of the repeat 44 (see Table 2).

**Figure 2:**
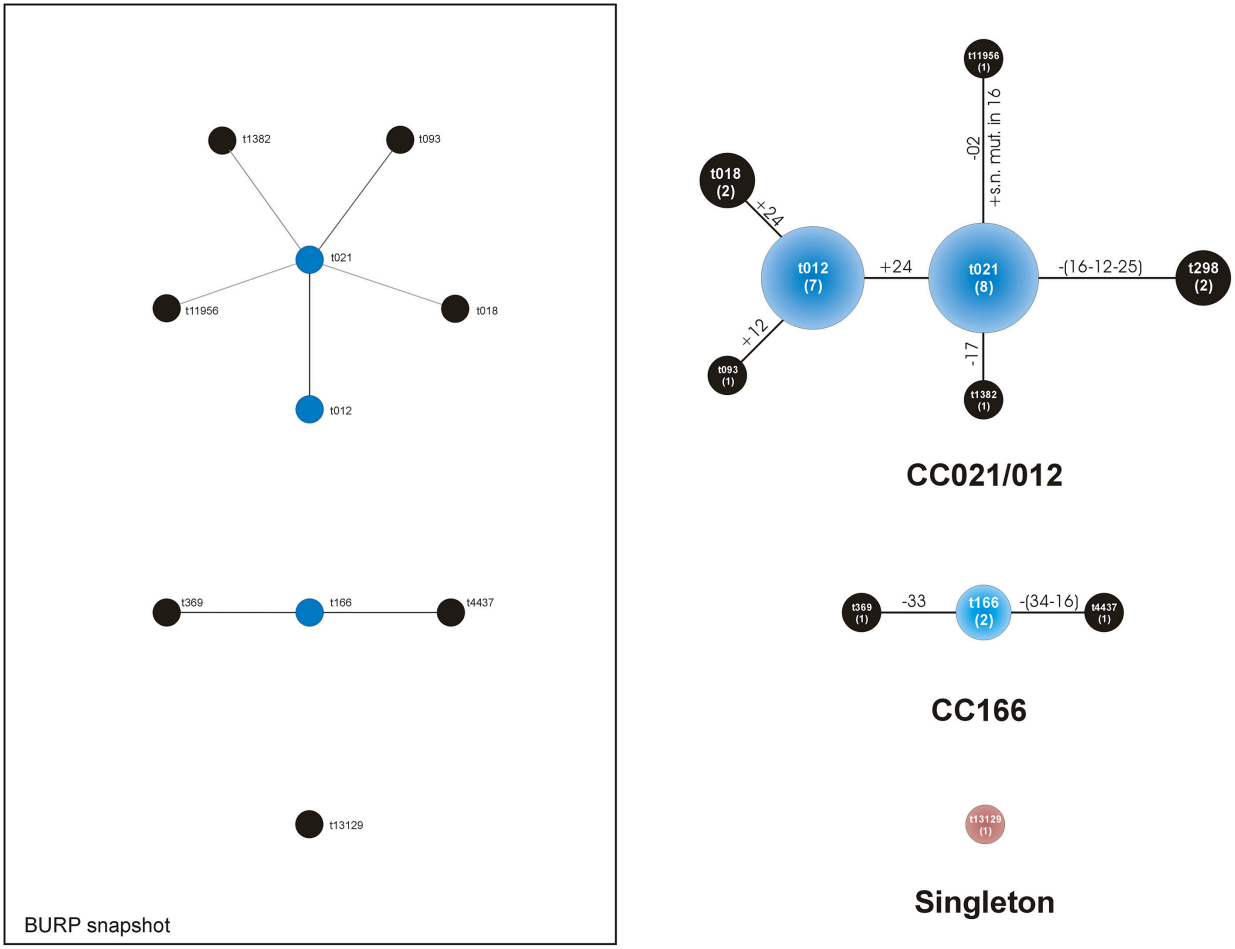
**Analysis of the *spa* types clustered by BURP algorithm of the Ridom StaphType Software**. On the left, within the frame, the population distribution is illustrated as per BURP population snapshot. The predict founder of a cluster is shown in blue, while the others in black. On the right, a customized representation of the CCs takes into consideration the type of mutations. Near the lines of connection, the mutations involved in the transition from a *spa* type to the next one are reported in detail. All DNA changes are meant to occur from the founder to the periphery. Legend: numbers along the lines refer to the repeat sequence involved in the mutation; + indicates the acquisition of a repeat sequence; -indicates the loss of a repeat sequence; within circles the numbers of the strains of each CC appear between brackets; extensive losses including more than a repeat sequence are reported along the lines between brackets; s.n. mut.: single nucleotide mutation of a sequence repeat.

The population structure analysis is illustrated in Figure 2, which reports the population analysis as per BURP population snapshot. On the right side of Figure 2 an additional drawing shows the type of mutations involved in the transition between *spa* types.

As far as the adhesin profile is concerned, differences were observed between the two main *spa*-CCs. The *spa*-CC021/012, including the vast majority of the strains of the ribocluster (81%), exhibited a remarkable prevalence of clinical strains endowed with both *cna* and *bbp* genes. Indeed, although the co-occurrence of *bbp* and *cna* genes was a genetic pattern consistently observed in the ribocluster (74%), its prevalence reached 91% within the *spa*-CC021/012. The prevalent *spa* type of CC021/012 was t021, uniquely consisting of *bbp*- and *cna*-positive strains. The triple adhesin gene pattern *bbp-cna-fib* was only observed in 7 strains belonging to *spa*-CC021/012 (32%).

All four strains of *spa*-CC166 (15% of the entire ribocluster) were free of *bbp* gene and, thus, lacked the combination *bbp*-*cna* (Tables 3, 4).

The *pvl*-positive ST243 *cra*2380 strain belonged to t021 and was *tst*-negative, positive for the tandem *bbp-cna* and susceptible to all the antibiotics tested. The overall prevalence of *tst* gene was 85% (23/27) and the only four strains without *tst* genes all belonged to the *spa*-CC021/012.

### Antibiotic resistance

Table 2S reports the observed prevalence of antibiotic resistance in the cluster of 27 strains. Apart from the frequent resistance to penicillin observed in 89% of the strains, the clinical strains were found to be nearly all sensitive to the rest of the antibiotics. Indeed, for the other 12 antibiotics, the number of resistant strains varied between 0 and 1 (4%). This profile of low antibiotic resistance was documented by the average multiple antibiotic resistance (MAR) index (Krumperman, 1983; Kaspar et al., 1990), which was as low as 0.24. Three strains were susceptible to all the antibiotics tested.

## Discussion

The principal outcome of this work is that the ribocluster initially interpreted as a clone (Campoccia et al., 2009) actually corresponds to a clonal complex, namely the MLST CC30, in which many organisms and diverse sequence types are grouped (Table 2). This finding highlights the higher discriminatory power of MLST and *spa*-typing with respect to ribotyping. Within CC30, all the strains were methicillin-susceptible and 93% of them belonged to ST30. In contrast to some STs, which are geographically concentrated, ST30 is spread worldwide (Vandenesch et al., 2003; DeLeo et al., 2010). MSSA isolates associated with CC30, particularly the ST30 genetic lineage, are responsible for various infections in different countries of the world (Robinson and Enright, 2003; Vandenesch et al., 2003; Aires de Sousa et al., 2005; Gomes et al., 2006; Vivoni et al., 2006; Hallin et al., 2007; Holtfreter et al., 2007; Fenner et al., 2008; Strommenger et al., 2008; He et al., 2013; Tavares et al., 2014). With regard to the CC30 clone in orthopedic infections, Aamot et al. (2012) reported that CC30 was the most frequent clonal complex in MSSA isolates from surgical site infections in orthopedic patients from Norway. MSSA isolates from orthopedic implant-related infections of Swiss and French hospitals, mainly belonging to the ST30-CC30, were highlighted by Post et al. (2014). Rincón et al. (2013) analyzed numerous isolates obtained from osteomyelitis in South American hospitals and described a high percentage of MSSA belonging to different genetic lineages, including ST30.

The absence of *mecA* and IS*256* (Tables 3, 4) reaffirms that antibiotic-resistance and hypervirulence (Benson et al., 2014) are not strictly necessary for the success of *S. aureus* lineages within CC30 and that other determinants might play a more relevant role, conferring greater fitness, and efficiency in host colonization and invasion (Ziebuhr et al., 1999; Arciola et al., 2002, 2004, 2015; Kiem et al., 2004; Valle et al., 2007; McAdam et al., 2012; Cheung et al., 2014; Lin et al., 2015).

In the Bayesian phylogenetic reconstruction of the CC30 lineage, presented in a recent work of McAdam et al. (2012), many of the CC30 related to hospital acquired EMRSA-16 exhibited the same genetic properties of the isolates of the present study. McAdam et al. (2012) showed that *tst* gene is absent in the isolates of the pandemic phage type 80/81 and SWP lineages, while present in 83% of isolates from the HA-EMRSA-16 lineage (ST36). Moreover, while phage type 80/81 and SWP clones were *pvl*-positive with a prevalence of 90%, EMRSA-16 and the other epidemic CC30 were all *pvl*-negative.

In our results, we observed the absence of *pvl* in all strains except one and 85% prevalence for *tst*. It is likely that MSSA-CC30 strains, especially those harboring *tst*, were strictly associated with the contemporary epidemic CC30 and related to the hospital acquired EMRSA-16 clone. In contrast to phage type 80/81 and SWP clones, the other epidemic CC30 related to EMRSA-16 appeared to be restricted to hospital settings and had reduced virulence, due in part to the low levels of expression of toxins such as PVL and LukE/LukD (Campoccia et al., 2008). McGavin et al. (2012) suggested that clade 3, comprising the contemporary hospital-associated MSSA-CC30 clone, brought a large burden of diseases due to its ability to persist in human hosts at the expense of an attenuated virulence. With regard to the strain *cra*2380, it turned out *pvl*-positive and *tst*-negative; this strain belongs to exactly the same MLST allelic profile (ST243) and *spa* type (t021) of the ATCC25923 reference strain. As reported by Chen et al. (2013), the ATCC25923 strain, isolated in USA (WA) in 1945, is a PVL-positive (PVL haplotype: H1a) CA-MSSA clinical strain belonging to the phage type 80/81 lineage.

The analysis of *spa* sequences by BURP algorithm allowed identification of two *spa* clonal complexes (CC021/012, CC166) and a singleton. As in the work of Wiśniewska et al. (2014), *spa*-CC021/012, with the main type t021 related to CC30, was the most frequent genetic lineage among our strains. CC166 strains and the singleton strain were all found to belong to the sequence type ST34 and exhibited some characteristic traits such as the absence of *cna*, a gene otherwise frequent within the predominant *spa*-CC021/012. *S. aureus* ST34 genetic background has been suggested to be of hybrid origin, being derived from recombination of large, contiguous portions of the chromosomes of genetically distinct parent backgrounds, with only part of the genome coming from CC30 (Robinson and Enright, 2004; Thomas et al., 2012). This notion may explain why ST34 lineage lacks the *bbp*-*cna* gene combination and may also provide an explanation why these strains belong to a different *spa* type. With the exclusion of the strains of the ST34 lineage, 91% of the remaining 22 strains possessed the *bbp*-*cna* gene combination, encoding for a couple of adhesins able to bind the most abundant bone proteins and crucial in the pathogenesis of orthopedic implant infections (Campoccia et al., 2009).

Moreover, all the strains shared the *agr type III* polymorphism. It should be remarked that strains with the same *agr* tend to be characterized by a well-defined pattern of virulence genes generated by intraspecific cross-inhibitions (Goerke et al., 2003).

In Tables 2–4, the MSSA ST30-t012 and ST30-t021 genetic patterns emerged as the two most prevalent *spa* repeat successions t021, differing from t012 in only one repeat sequence, indicating that they are likely to be close relatives. Holtfreter et al. (2007) showed that in MSSA-CC30 isolates, the *spa* type t012 was the most prevalent among nasal isolates, while the *spa* type t021 was most prevalent among blood culture isolates. In Denmark, in a study of MSSA clinical isolates from blood cultures, the authors presented ST30-t021 as the most frequent genetic lineage (Gomes et al., 2006). Fenner et al. also observed t021 and t012 in invasive MSSA (Fenner et al., 2008) in a Swiss University Hospital, as did Nulens et al. (2008) in bloodstream isolates collected from a Dutch university hospital. Post et al. (2014) indicated the presence of *spa* type t012 (ST30) as the most frequent among the MSSA isolates.

The most prevalent clonal type ST30-t012 of our collection has also been recurrently observed in Portugal, Spain, Belgium, the Netherlands and in the USA, by analyzing MSSA isolates from different time periods (Aires de Sousa et al., 2005; Rijnders et al., 2009; Argudín et al., 2013; Miko et al., 2013; Tavares et al., 2014). In other countries, such as China, Taiwan, the African countries of Cameroon, Madagascar, Senegal, Niger and Morocco, MSSA genetic lineages from community infections were dissimilar (Rijnders et al., 2009; Breurec et al., 2011; He et al., 2013). The reason for the differences in MSSA lineages among various countries still remains not well understood. Also the discovery of a new *spa* type (t13129) and of two new sequence types (ST2954, ST2960) suggests that there is still much to be revealed about the evolution of CC30. The success of CC30 could be multifactorial and its panel of adhesins represents a successful strategy for renewing and continuing its adaptation to different niches.

### Conflict of interest statement

The authors declare that the research was conducted in the absence of any commercial or financial relationships that could be construed as a potential conflict of interest.

## Footnotes

^1^Center for Disease Control and Prevention (CDC). Available online at http://www.cdc.gov/

^2^Ridom SpaServer database. Available online at http://www.spaserver.ridom.de

^3^MLST website. Available online at http://saureus.mlst.net/sql/multiplelocus.asp

^4^MLST database. Available online at http://www.mlst.net

^5^eBURST algorithm. Available online http://eburst.mlst.net/v3/mlst_datasets/
