# Supplementary material for: Molecular Characterization of a Prevalent Ribocluster of Methicillin-Sensitive Staphylococcus aureus from Orthopedic Implant Infections. Correspondence with MLST CC30
Source: Front Cell Infect Microbiol. 2016 Feb 16;6:8. doi: 10.3389/fcimb.2016.00008 (PMC4754407; doi:10.3389/fcimb.2016.00008)
Supplement: Supplementary file 1 [file Table1.doc]

**Table 1S. Biographical-evolutionary sketch of *S. aureus* CC30 and association with diseases**

| ***S. aureus* CC30 biographical sketch** | | |
| --- | --- | --- |
| **Per-iod** | **Observations** | **References** |
| 1950s | *S. aureus* clonal complex 30 (CC30) recognized as responsible for infectious epidemics, three pandemic “waves”, and toxic shock syndrome (TSS) epidemics. | McAdam et al., 2012; Chen et al., 2012; Chambers and Deleo, 2007; Rountree and Beard, 1958 |
| 1953 | First CC30 pandemic by an MSSA clone, known as phage-type 80/81, isolated in neonatal infections in Australia, characterized by production of Panton-Valentine leukocidin toxin and resistance to penicillin | Hassall and Rountree, 1959 |
| 1953-1963 | The pandemic lasted ten years (1953-1963), at first spreading in healthcare environments, then in the community and declining following the introduction of methicillin | DeLeo et al., 2011 |
| 1959 | Methicillin licensed in Great Britain |  |
| 1960s | First detection of MRSA in Australia | Deurenberg and Stobberingh, 2008 |
| 1967-1975 | Identification of other CC30 lineages, responsible for worldwide diseases: (i) the hyper-virulent Southwest Pacific (SWP) clone; (ii) the contemporary hospital-associated MRSA (HA-MRSA) clone EMRSA-16 and (iii) its related methicillin-susceptible *S. aureus* (MSSA) epidemic lineage (Con). | Wiśniewska et al., 2014; Chen et al., 2012 |
| 1842-1960 | 1842 (1765-1909) estimated date of emergence of the most recent common ancestor (MRCA) of the entire CC30 lineage. Estimated dates of emergence of the three major MRCA clades, corresponding to the three pandemic CC30 clones: 1936 (1926-1945) for phage 80/81, 1967 (1952-1984) for SWP, 1975 (1965-1983) for EMRSA-16. 1950-60 estimated date of emergence of the other contemporary epidemic MRSA/MSSA CC30 related to EMRSA-16 clone | McAdam et al., 2012 |
| 1960s | Origin of MRSA by transfer of SCC*mec* element into existing MSSA lineages with a genetic background similar to that of MRSA clones, including CC30. | Hallin et al., 2007; Nulens et al., 2008; Brady et al., 2007 |
| 1980s | MSSA-ST30 acquired in a first time *pvl* and then SCC*mec* genes to become a PVL^+^ MRSA | David et al., 2010; Diep et al., 2006 |
| 1980 | Description of an ST30 strain, carrying the SCC*mec* type IV element but deficient of *pvl*, possibly representing a precursor of the PVL^+^ CA-MRSA. Difficulty to distinguish between CA- and HA-MSSA and changes in the epidemiology of both MRSA and MSSA. | Larsen et al., 2012; David et al., 2010; Coombs et al., 2004 |
| 1993 | First CA-MRSA episode, when CC30-ST30 clone emerged in Western Australia associated to the Southwest Pacific (SWP) clone (CC30-ST30-SCC*mec*IV and *pvl*-positive) | Nimmo et al., 2000; Munckhof et al., 2003; Robinson et al., 2005 |
|  | | |
| ***S. aureus* CC30 evolutionary characteristics and association with diseases** | | |
| MSSA has in general more genetic lineages than MRSA, suggesting more heterogeneity | | Strommenger et al., 2008; Brady et al., 2007 |
| MSSA clinical isolates have wide genetic diversity across different clinical circumstances, locations or time periods. | | Miko et al., 2013 |
| CC30 is one of the most prominent MSSA clones | | Post et al., 2014; He et al., 2013; Vandenesch et al., 2003; Hallin et al., 2007; Robinson and Enright, 2003; Aires de Sousa et al., 2005; Gomes et al., 2006; Vivoni et al., 2006; Holtfreter et al., 2007; Fenner et al., 2008; Tavares et al., 2014   Robinson et al., 2005 |
| MSSA isolates associated with CC30 has been described responsible for diverse sites of infection in different world countries, in particular with the ST30 genetic lineage. | | He et al., 2013; Hallin et al., 2007; Strommenger et al., 2008; Robinson and Enright, 2003; Aires de Sousa et al., 2005; Gomes et al., 2006; Vivoni et al., 2006; Holtfreter et al., 2007; Fenner et al., 2008; Tavares et al., 2014 |
| CC30 has been found prevalent among MSSA isolates from nasal carriage, uncomplicated infections, and bacteremias with hematogenous complications, | | Larsen et al., 2012; Robinson et al., 2005 ; Fowler et al., 2007 |
| Evidence of clonal association between MSSA-CC30 and *tst* in nasal carriage and bacteremia and in infective endocarditis. A high prevalence of MSSA CC30 isolates in bloodstream infections and infective endocarditis. | | Robinson and Enright, 2003; Holtfreter et al., 2007; Campanile et al., 2012; Nienaber et al., 2011 |
| Evidence of clonal association between MSSA-CC30 and a large spectrum of infections, including osteomyelitis and orthopedic-associated infections. | | Post et al., 2014; Aamot et al., 2012; Rincón et al., 2013; Hennig and Ziebuhr, 2010 |
| Sequence type 30 (ST30), as established through MLST analysis, was the genetic founder of CC30. MSSA isolates of phage 80/81 and Con lineage were for the majority ST30 and *spa* type t021 | | Aires de Sousa et al., 2005; Chen et al., 2012; Nimmo et al., 2000; Robinson et al., 2005 |
| A correlation between ST30 and t021 or t012 has been shown. | | Post et al., 2014; David et al., 2010; Chen et al., 2013; DeLeo et al., 2011 |
| The contemporary hospital-associated MSSA-CC30 clone, found to be ST30-t012, frequently encodes toxic shock syndrome toxin, exhibits an attenuated virulence in murine infection models and has a large burden of diseases. | | Cheung et al., 2014; McGavin et al., 2012 |
| The contemporary CC30 isolates, which are often methicillin-susceptible, have no genes codifying for Panton-Valentine leukocidin and are abundant in *tst* gene. | | McAdam et al., 2012 |
| Hospital-acquired *S. aureus* has a larger panel of antibiotic-resistances and a limited toxin potential. The minor expression of cytolytic toxins in contemporary CC30, when compared to historic clones, appears interconnected to the fact that contemporary CC30 causes hospital-acquired infections primarily. | | DeLeo et al., 2011 |
| In contemporary CC30 clones, mutations of phenol-soluble modulin α (PSMα) and α-hemolysin (*hla*), leading to lower pro-inflammatory and cytolytic potential, play a role for the adaptation to hospital-associated infections, allowing pathogens to evade the immune system and to achieve long-term colonization and persistence in human host. | | Cheung et al., 2014 |
| MRSA, although considered the major HA-MRSA, has been reported as the etiologic agent of infections in healthy people in the community (CA-MRSA). CA-MRSA is different from HA-MRSA since it is commonly susceptible to a large spectrum of antibiotics besides β-lactams, endowed with types IV and V SCC*mec*, and often associated with the presence of *pvl*. | | DeLeo et al., 2011 |
| IS*256* has been recognized as a typical marker for hospital-acquired multi-resistant isolates | | Munckhof et al., 2003; Hennig and Ziebuhr, 2010 |
| IS*256* negative isolates could have a hypo-virulence profile, but a biofilm-positive phenotype, an important virulence factor in implant-related infection. | | Ziebuhr et al., 1999; Arciola et al., 2004; Kiem et al., 2004; Arciola et al., 2015; Arciola et al., 2002 ; Lin et al., 2015 |

**REFERENCES**

Aamot, H. V., Blomfeldt, A., Skråmm, I., Müller, F., and Monecke, S. (2012). Molecular characterisation of methicillin-sensitive *Staphylococcus aureus* from

deep surgical site infections in orthopaedic patients. *Eur. J. Clin. Microbiol. Infect. Dis*. 31, 1999–2004. doi: 10.1007/s10096-011-1532-3

Aires de Sousa, M., Conceição, T., Simas, C., and de Lencastre, H. (2005).Comparison of genetic backgrounds of methicillin-resistant and –susceptible *Staphylococcus aureus* isolates from Portuguese hospitals and the community. *J. Clin. Microbiol*. 43, 5150–5157. doi: 10.1128/JCM.43.10.5150-5157.2005

Arciola, C. R., Baldassarri, L., and Montanaro, L. (2002). In catheter infections by *Staphylococcus epidermidis* the intercellular adhesion (*ica*) locus is a molecular marker of the virulent slime-producing strains. *J. Biomed. Mater. Res*. 59, 557–562. doi: 10.1002/jbm.10006

Arciola, C. R., Campoccia, D., Gamberini, S., Rizzi, S., Donati, M. E., Baldassarri, L., et al. (2004). Search for the insertion element IS*256* within the *ica* locus of *Staphylococcus epidermidis* clinical isolates collected from biomaterial-associated infections. *Biomaterials* 25, 4117–4125. doi: 10.1016/j.biomaterials.2003.11.027

Arciola, C. R., Campoccia, D., Ravaioli, S., and Montanaro, L. (2015). Polysaccharide intercellular adhesin in biofilm: structural and regulatory aspects. *Front. Cell. Infect. Microbiol*. 5:7. doi: 10.3389/fcimb.2015.00007

Brady, J. M., Stemper, M. E., Weigel, A., Chyou, P. H., Reed, K. D., and Shukla, S. K. (2007). Sporadic “transitional” community-associated methicillin-resistant *Staphylococcus aureus* strains from health care facilities in the United States. *J. Clin. Microbiol.* 45, 2654–2661. doi: 10.1128/JCM.02579-06

Campanile, F., Bongiorno, D., Falcone, M., Vailati, F., Pasticci, M. B., Perez, M., et al. (2012). Changing Italian nosocomial-community trends and heteroresistance in *Staphylococcus aureus* from bacteremia and endocarditis. *Eur. J. Clin.Microbiol. Infect. Dis.* 31, 739–745. doi: 10.1007/s10096-011-1367-y

Chambers, H. F., and Deleo, F. R. (2007). Waves of resistance: *Staphylococcus aureus* in the antibiotic era. *Nat. Rev. Microbiol.* 7, 629–641. doi: 10.1038/nrmicro2200

Chen, F. J., Siu, L. K., Lin, J. C., Wang, C. H., and Lu, P. L. (2012). Molecular typing and characterization of nasal carriage and community-onset infection methicillin-susceptible *Staphylococcus aureus* isolates in two Taiwan medical centers. *BMC Infect. Dis.* 12:343. doi: 10.1186/1471-2334-12-343

Chen, L., Chavda, K. D., Solanki, M., Mediavilla, J. R., Mathema, B., Schlievert, P. M., et al. (2013). Genetic variation among Panton-Valentine leukocidin encoding bacteriophages in *Staphylococcus aureus* clonal complex 30 strains. *J. Clin. Microbiol.* 51, 914–919. doi: 10.1128/JCM.03015-12

Cheung, G. Y., Kretschmer, D., Duong, A. C., Yeh, A. J., Ho, T. V., Chen, Y., et al. (2014). Production of an attenuated phenol-soluble modulin variant unique to the MRSA clonal complex 30 increases severity of bloodstream infection. *PLoS Pathog.* 10:e1004298. doi: 10.1371/journal.ppat.1004298

Coombs, G.W., Nimmo, G. R., Bell, J.M., Huygens, F., O’Brien, F. G.,Malkowski, M. J., et al. (2004). Genetic diversity among community methicillin-resistant *Staphylococcus aureus* strains causing outpatient infections in Australia. *J. Clin. Microbiol.* 42, 4735–4743. doi: 10.1128/JCM.42.10.4735-4743.2004

David, M. Z., and Daum, R. S. (2010). Community-associated methicillin resistant *Staphylococcus aureus*: epidemiology and clinical consequences of an emerging epidemic. *Clin. Microbiol. Rev*. 23, 616–687. doi: 10.1128/CMR.00081-09

DeLeo, F. R., Kennedy, A. D., Chen, L., Bubeck Wardenburg, J., Kobayashi, S. D., Mathema, B., et al. (2011). Molecular differentiation of historic phage-type 80/81 and contemporary epidemic *Staphylococcus aureus*. *Proc. Natl. Acad. Sci. U.S.A*. 108, 18091–18096. doi: 10.1073/pnas.1111084108

Deurenberg, R. H., and Stobberingh, E. E. (2008). The evolution of *Staphylococcus aureus*. *Infect. Genet. Evol*. 8, 747–763. doi: 10.1016/j.meegid.2008.07.007

Diep, B. A., Carleton, H. A., Chang, R. F., Sensabaugh, G. F., and Perdreau- Remington, F. (2006). Roles of 34 virulence genes in the evolution of hospital and community-associated strains of methicillin-resistant *Staphylococcus aureus*. *J. Infect. Dis*. 193:1495–1503. doi: 10.1086/503777

Fenner, L.,Widmer, A. F., and Frei, R. (2008). Molecular epidemiology of invasive methicillin-susceptible *Staphylococcus aureus* strains circulating at a Swiss

University Hospital. *Eur. J. Clin. Microbiol.* Infect. Dis. 27, 623–626. doi: 10.1007/s10096-008-0463-0

Gomes, A. R., Westh, H., and de Lencastre, H. (2006). Origins and evolution of methicillin-resistant *Staphylococcus aureus* clonal lineages. *Antimicrob. Agents Chemother*. 50, 3237–3244. doi: 10.1128/AAC.00521-06

Hallin, M., Denis, O., Deplano, A., DeMendonça, R., De Ryck, R., Rottiers, S., et al. (2007). Genetic relatedness between methicillin-susceptible and methicillin-resistant *Staphylococcus aureus*: results of a national survey. *J. Antimicrob. Chemother*. 59, 465–472. doi: 10.1093/jac/dkl535

Hassall, J. E., and Rountree, P. M. (1959). Staphylococcal septicaemia. *Lancet* 1, 213–217. doi: 10.1016/S0140-6736(59)90047-9

He, W., Chen, H., Zhao, C., Zhang, F., Li, H., Wang, Q., et al. (2013). Population structure and characterisation of *Staphylococcus aureus* from bacteraemia at multiple hospitals in China: association between antimicrobial resistance, toxin genes and genotypes. *Int. J. Antimicrob. Agents* 42, 211–219. doi:10.1016/j.ijantimicag.2013.04.031

Hennig, S., and Ziebuhr, W. (2010). Characterization of the transposase encoded by 17, the prototype of a major family of bacterial insertion sequence elements. *J. Bacteriol*. 192, 4153–4163. doi: 10.1128/JB.00226-10

Holtfreter, S., Grumann, D., Schmudde, M., Nguyen, H. T., Eichler, P., Strommenger, B., et al. (2007). Clonal distribution of superantigen genes in clinical *Staphylococcus aureus* isolates*. J. Clin. Microbiol*. 45, 2669–2680. doi:10.1128/JCM.00204-07

Kiem, S., Oh,W. S., Peck, K. R., Lee, N. Y., Lee, J. Y., Song, J. H., et al. (2004). Phase variation of biofilm formation in *Staphylococcus aureus* by IS*256* insertion and its impact on the capacity adhering to polyurethane surface. *J. KoreanMed. Sci*.19, 779–782. doi: 10.3346/jkms.2004.19.6.779

Lin, M. H., Shu, J. C., Lin, L. P., Chong, K. Y., Cheng, Y.W., Du, J. F., et al. (2015). Elucidating the crucial role of poly N-acetylglucosamine from *Staphylococcus aureus* in cellular adhesion and pathogenesis. *PLoS ONE* 10:e0124216. doi:10.1371/journal.pone.0124216

McAdam, P. R., Templeton, K. E., Edwards, G. F., Holden, M. T., Feil, E. J., Aanensen, D. M., et al. (2012). Molecular tracing of the emergence, adaptation, and transmission of hospital-associated methicillin-resistant *Staphylococcus aureus*. *Proc. Natl. Acad. Sci. U.S.A*. 109, 9107–9112. doi:10.1073/pnas.1202869109

McGavin, M. J., Arsic, B., and Nickerson, N. N. (2012). Evolutionary blueprint for host- and niche-adaptation in *Staphylococcus aureus* clonal complex CC30. *Front. Cell. Infect. Microbiol*. 2:48. doi: 10.3389/fcimb.2012.00048

Miko, B. A., Hafer, C. A., Lee, C. J., Sullivan, S. B., Hackel, M. A., Johnson, B. M., et al. (2013). Molecular characterization of methicillin-susceptible *Staphylococcus aureus* clinical isolates in the United States, 2004 to 2010. *J. Clin. Microbiol.* 51, 874–879. doi: 10.1128/JCM.00923-12

Munckhof, W. J., Schooneveldt, J., Coombs, G. W., Hoare, J., and Nimmo, G. R. (2003). Emergence of community-acquired methicillin-resistant *Staphylococcus aureus* (MRSA) infection in Queensland, Australia. *Int. J. Infect. Dis*. 7, 259–264. doi: 10.1016/S1201-9712(03)90104-4

Nienaber, J. J., Sharma Kuinkel, B. K., Clarke-Pearson, M., Lamlertthon, S., Park, L., Rude, T. H., et al. (2011). International-collaboration on endocarditis microbiology investigators. Methicillin-susceptible *Staphylococcus aureus* endocarditis isolates are associated with clonal complex 30 genotype and a distinct repertoire of enterotoxins and adhesins. *J. Infect. Dis.* 204, 704–713. doi: 10.1093/infdis/jir389

Nimmo, G. R., Schooneveldt, J., O’Kane, G., McCall, B., and Vickery, A. (2000). Community acquisition of gentamicin-sensitive methicillin-resistant *Staphylococcus aureus* in southeast Queensland, Australia. *J. Clin.Microbiol.* 38, 3926–3931.

Nulens, E., Stobberingh, E. E., van Dessel, H., Sebastian, S., van Tiel, F. H., Beisser, P. S., et al. (2008). Molecular characterization of *Staphylococcus aureus* blood stream isolates collected in a Dutch University Hospital between 1999 and 2006. *J. Clin. Microbiol*. 46, 2438–2441. doi: 10.1128/JCM.00808-08

Post, V., Wahl, P., Uçkay, I., Ochsner, P., Zimmerli, W., Corvec, S., et al. (2014). Phenotypic and genotypic characterisation of *Staphylococcus aureus* causing musculoskeletal infections. *Int. J. Med. Microbiol.* 304, 565–576. doi:10.1016/j.ijmm.2014.03.003

Rincón, S., Reyes, J., Carvajal, L. P., Rojas, N., Cortés, F., Panesso, D., et al. (2013). Cefazolin high-inoculum effect in methicillin-susceptible *Staphylococcus aureus* from South American hospitals. *J. Antimicrob. Chemother.* 68, 2773–2778. doi: 10.1093/jac/dkt254

Robinson, D. A., and Enright,M. C. (2003). Evolutionary models of the emergence of methicillin-resistant *Staphylococcus aureus*. *Antimicrob. Agents Chemother.* 47, 3926–3934. doi: 10.1128/AAC.47.12.3926-3934.2003

Robinson, D. A., Kearns, A. M., Holmes, A., Morrison, D., Grundmann, H., Edwards, G., et al. (2005). Re-emergence of early pandemic *Staphylococcus aureus* as a community-acquired methicillin-resistant clone. *Lancet* 365, 1256–1258. doi: 10.1016/S0140-6736(05)74814-5

Rountree, P. M., and Beard, M. A. (1958). Further observations on infection with phage type 80 Staphylococci in Australia. *Med. J. Aust.* 45, 789–795.

Strommenger, B., Braulke, C., Heuck, D., Schmidt, C., Pasemann, B., Nübel, U., et al. (2008). *spa* Typing of *Staphylococcus aureus* as a frontline tool in epidemiological typing. *J. Clin. Microbiol.* 46, 574–581. doi:10.1128/JCM.01599-07

Tavares, A., Faria, N. A., de Lencastre, H., and Miragaia, M. (2014). Population structure of methicillin-susceptible *Staphylococcus aureus* (MSSA) in Portugal over a 19-year period (1992-2011). *Eur. J. Clin. Microbiol. Infect. Dis.* 33, 423–432. doi: 10.1007/s10096-013-1972-z

Vandenesch, F., Naimi, T., Enright, M. C., Lina, G., Nimmo, G. R., Heffernan, H., et al. (2003). Community-acquired methicillin-resistant *Staphylococcus* *aureus* carrying Panton-Valentine leukocidin genes: worldwide emergence. *Emerg. Infect. Dis*. 9, 978–984. doi: 10.3201/eid0908.030089

Vivoni, A. M., Diep, B. A., de Gouveia Magalhães, A. C., Santos, K. R., Riley, L. W., Sensabaugh, G. F., et al. (2006). Clonal composition of *Staphylococcus aureus* isolates at a Brazilian university hospital: identification of international circulating lineages. *J. Clin. Microbiol.* 44, 1686–1691. doi:10.1128/JCM.44.5.1686-1691.2006

Wiśniewska, K., Piórkowska, A., Kasprzyk, J., Bronk, M., and Swie´c, K. (2014). Clonal distribution of bone sialoprotein-binding protein gene among *Staphylococcus aureus* isolates associated with bloodstream infections. *Folia Microbiol. (Praha).* 59, 465–471. doi: 10.1007/s12223-014-0321-7

Ziebuhr,W., Krimmer, V., Rachid, S., Lössner, I., Götz, F., and Hacker, J. (1999). A novel mechanism of phase variation of virulence in *Staphylococcus epidermidis*: evidence for control of the polysaccharide intercellular adhesin synthesis by alternating insertion and excision of the insertion sequence element IS*256*.*Mol. Microbiol.* 32, 345–356. doi: 10.1046/j.1365-2958.1999.01353.x
