# Supplementary material for: Molecular Characterization of a Prevalent Ribocluster of Methicillin-Sensitive Staphylococcus aureus from Orthopedic Implant Infections. Correspondence with MLST CC30
Source: Front Cell Infect Microbiol. 2016 Feb 16;6:8. doi: 10.3389/fcimb.2016.00008 (PMC4754407; doi:10.3389/fcimb.2016.00008)
Supplement: Supplementary file 2 [file Table2.doc]

**Table 2S.** Overall prevalence of phenotypic antibiotic resistance

|  | **OXA** | **IPM** | **PEN** | **AMP** | **CFZ** | **FAM** | **GEN** | **AMK** | **NET** | **TOB** | **ERY** | **CLI** | **CHL** | **SXT** | **CIP** | **VAN** |
| --- | --- | --- | --- | --- | --- | --- | --- | --- | --- | --- | --- | --- | --- | --- | --- | --- |
| Resistant strains (Percent) | 0  (0%) | 1  (4%) | 24  (89%) | 24  (89%) | 24  (89%) | 24  (89%) | 1  (4%) | 1  (4%) | 1  (4%) | 1  (4%) | 1  (4%) | 0  (0%) | 0  (0%) | 0  (0%) | 1  (4%) | 0  (0%) |

The average MAR value for the 27 strains was 0.24. Legend: oxacillin (OXA); imipenem (IPM); penicillin (PEN); AMP (ampicillin); cefazolin (CFZ); cefamandole (FAM); gentamicin (GEN); amikacin (AMK); netilmicin (NET); tobramycin (TOB); erythromycin (ERY); clindamycin (CLI); chloramphenicol (CHL); trimethoprim-sulfamethoxazole (SXT); ciprofloxacin (CIP); vancomycin (VAN)
